# Prospective Approaches to the Sustainable Use of Peonies in Bulgaria

**DOI:** 10.3390/plants14060969

**Published:** 2025-03-19

**Authors:** Christina Stoycheva, Daniela Batovska, Giuseppe Antonio Malfa, Rosaria Acquaviva, Giancarlo Statti, Ekaterina Kozuharova

**Affiliations:** 1 Department of Pharmacognosy, Faculty of Pharmacy, Medical University-Sofia, 1000 Sofia, Bulgaria; christina9828@gmail.com; 2 Institute of Engineering Chemistry, Bulgarian Academy of Sciences, Acad. G. Bonchev, Bl. 103, 1113 Sofia, Bulgaria; danielabatovska@gmail.com; 3 Department of Drug and Health Sciences, University of Catania, Viale A. Doria 6, 95125 Catania, Italy; gmalfa@unict.it (G.A.M.); racquavi@unict.it (R.A.); 4 Research Centre on Nutraceuticals and Health Products (CERNUT), University of Catania, Viale A. Doria 6, 95125 Catania, Italy; 5 Department of Pharmacy, Health Sciences and Nutrition, University of Calabria, Via P. Bucci, 87030 Rende, Italy; giancarlo.statti@unical.it

**Keywords:** *Paeonia peregrina*, *P. officinalis*, *P. tenuifolia*, *P. mascula*, *P. lactiflora*, chemical composition, biological activity, pharmacological effects

## Abstract

In Europe, *Paeonia officinalis* and *P. peregrina*, along with Chinese *P. lactiflora*, are commonly used for medicinal purposes. This comprehensive review summarizes the secondary metabolites and biological activities of *P. peregrina*, *P*. *officinalis*, *P. tenuifolia*, *P. mascula*, *P. lactiflora*, and the ornamental cultivars derived from the last taxon. Terpenoids, flavonoids, and phenolic acids are present in all five species, while tannins, lipids, and organic acids have been identified in only some. All five species exhibit antioxidant and antimicrobial potential, alongside anti-inflammatory, anticancer, neuroprotective, antisclerotic, antidiabetic, and various other bioactivities. The data were accessed via Scopus, Web of Science, PubMed, and Google Scholar search engines. The review also reveals that *P. officinalis* and *P. lactiflora* have been far more extensively studied than *P. peregrina*, *P. tenuifolia*, and *P. mascula* in terms of their chemical composition and pharmacological properties. The genus *Paeonia* L. comprises 37 accepted species, many of which are renowned for their ornamental and medicinal value. Native to Bulgaria are *P. peregrina*, *P. tenuifolia*, and *P. mascula*, with the latter two being protected by the Bulgarian Biodiversity Act. The collection of substances from all three species is subject to regulatory restrictions. This review reveals the possible use of *P. lactiflora* as a substitute for *P. peregrina.*

## 1. Introduction

The genus *Paeonia* L. consists of 37 accepted species [1]. Peonies are widely recognized as ornamental plants. They are also well known for their medicinal properties, being an integral part of traditional medicine in various cultures as well as their use in alternative medicine [2]. Several *Paeonia* species and hybrids have been utilized as traditional Chinese medicinal materials for over 2000 years to treat cardiovascular, extravasated blood, and female genital diseases [3,4]. In Europe, *Paeonia officinalis* L. [5,6,7,8,9] and *P. peregrina* Mill. are commonly used for medicinal purposes [10,11,12,13].

The European Medicines Agency (EMA) issues scientific opinions on herbal substances and preparations, along with information on recommended uses and safety conditions. Interestingly, concerning peonies, the EMA refers only to Chinese *P. lactiflora* Pallas [14] and *P. veitchii* Lynch [15], with a native range of distribution from Eastern Tibet to Northern and Central China [16].

Native to Bulgaria are *P. peregrina*, *P. tenuifolia* L., and *P. mascula* (L.) Mill., the last two taxa being rare plants protected by the Bulgarian Biological Diversity Act [17]. All activities that endanger the populations of *P. tenuifolia* and *P. mascula* (including picking, cutting, collecting, etc.) are strictly forbidden. The gathering of *P. peregrina* plant substances is under a restriction regime [18]. The annual harvest quantities of petals and roots from wild populations are regulated and monitored by the Ministry of Environment and Water of Bulgaria. The vulnerability of *P. peregrina* in its natural habitat motivates future efforts to introduce the species to cultivation or to substitute its plant materials with those derived from the widely cultivated ornamental species *P. lactiflora.* Consequently, an overview of peonies native to the Balkans, along with the ornamental *P. lactiflora*, is essential.

The traditional medicinal use of European peonies and the most popular Chinese one, namely *P. lactiflora*, are characterized by some specifics.

Various parts of *Paeonia officinalis* L., including its roots, seeds, and flowers, have been widely used in traditional herbal medicine in Spain, France, Italy, and the Northwestern Balkans [19,20,21] to address conditions such as gout, fever, and digestive issues, owing to their reported anti-inflammatory, antispasmodic, and analgesic properties [5]. Additionally, its calming effects have historically been used to treat certain nervous system disorders, including epilepsy [2,21].

**Figure 1:**
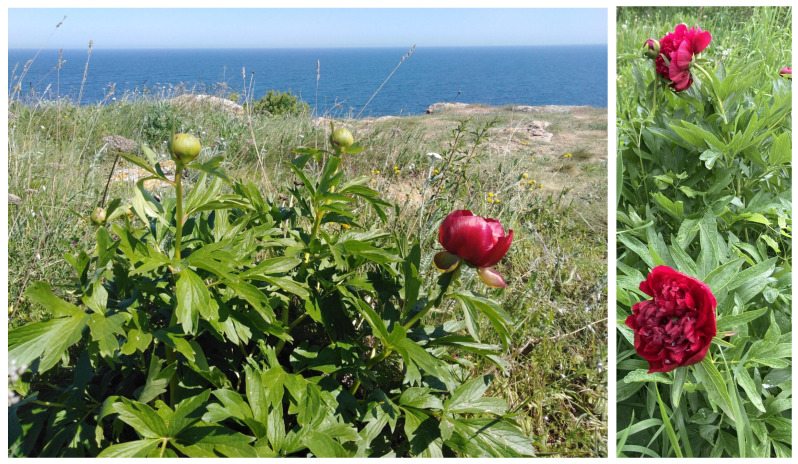
*Paeonia peregrina* in situ and ornamental *P. lactiflora* (photo E. Kozuharova).

*Paeonia peregrina* Mill. (Figure 1) is known and recommended for its analgesic, sedative, and anti-inflammatory properties, as well as for its use in treating female genital diseases and blood stagnation [22,23]. It is vastly used nowadays and, since natural resources are restricted, its gathering is controlled by the Ministry of Environment and Water [18].

Although the native range of *Paeonia mascula* (L.) Mill. extends from Northern Spain to Iraq [1,23], an infusion made from the roots has been traditionally used as an antihemorrhagic, antispasmodic, and sedative agent, as well as a remedy for coughs and sore throats, only in Izmir Province, Turkey [24].

*Paeonia tenuifolia* is called steppe peony because of its distribution [25,26], but little is published about its traditional medicinal use.

*Paeonia lactiflora* Pallas is known for its ornamental and medicinal value and is widely cultivated in Europe, Asia, and North America [25,26]. More than 500 cultivars of *P. lactiflora* are found in China, with numerous studies focused on their classification based on phylogenetics, phytochemistry, cultivation physiology, flower color, and more [27]. In the Pharmacopoeia of the People’s Republic of China (2010), two therapeutic herbal products derived from *P. lactiflora* are listed: Radix paeoniae alba (baishao or white peony root) and Radix paeoniae rubra (chishao or red peony root) [28]. Production methods allow *P. lactiflora* to be sold as white or red type (Figure 1). Usually, cultivated *P. lactiflora* is used for white peony root production, with its roots boiled and peeled before sun drying. For red peony root, wild sources of *P. lactiflora* are used, with the root bark left intact [29].

Peonies have a low germination rate, difficult to break seed dormancy, and long germination period. The in vitro seed culture of *Paeonia peregrina* from Bulgaria has failed so far in all experimental regimes, including scarification, stratification, and gibberellic acid (GA_3_) treatment [Stanilova personal communication]. The seeds of *P. peregrina* from four natural habitats in Serbia have an underdeveloped embryo that needs to grow inside the seed, which slows down the germination. Treatments with GA_3_ can promote the process [30]. Studies on the propagation of *P. peregrina*, either by seed or vegetatively, are insufficient, and thus the possibility of cultivation of this species is still dubious.

The aim of this study is to summarize the secondary metabolites and biological activities of *P. peregrina*, *P*. *officinalis*, *P. tenuifolia*, *P. mascula*, *P. lactiflora*, and the ornamental cultivars derived from the latter. As well, this study aims to evaluate the potential of using *P. lactiflora* as a substitute for *P. peregrina* based on the obtained data.

## 2. Results and Discussion

### 2.1. Bioactive Compounds of Peonies

The phytochemical composition of *P. peregrina*, *P*. *officinalis*, *P. tenuifolia*, *P. mascula*, and *P. lactiflora* is summarized in Table 1. The number of identified bioactive compounds in the studied peonies is 182. They belong to the following classes: terpenes (terpenoids), phenoloc compounds (flavonoids, phenolic acids, tannins, and other phenolic compounds), lipids, and organic acids. Additionally, one study has detected alkaloids (only oxindole and 5-hydroxyquinoline and only in *P. lactiflora*).

#### 2.1.1. Terpenoids

The presence of monoterpene glycosides is characteristic of all selected *Paeonia* species (Table 1), with paeoniflorin being the most abundant and extensively studied compound. The unique ‘cage-like’ pinane structure, attached to a sugar moiety, underpins the diverse pharmacological activities of paeoniflorin. It exhibits potent antioxidant, anti-inflammatory, antithrombotic, anticonvulsive, analgesic, cardioprotective, neuroprotective, hepatoprotective, antidepressant-like, antitumoral, and immune-regulatory effects [31,32].

Similar multiple activities have also been demonstrated for albiflorin, the functional isomer of paeoniflorin, along with their derivatives identified across the species. These compounds, with various modifications such as ester linkages to benzoic, *p*-hydroxybenzoic, and gallic acids, further enhance the therapeutic potential of *Paeonia* species [33]. The anti-inflammatory compound paeonidanin, with a skeleton similar to albiflorin, has been identified in *P. peregrina*, *P. mascula*, and *P. lactiflora* [29,34,35,36,37,38].

Other structurally diverse monoterpene glycosides have been identified as minor components across the five *Paeonia* species, but their low abundance has limited studies on their bioactivities. Paeonin B is present in *P. peregrina*, *P. tenuifolia*, and *P. lactiflora* [13,39,40]. Isomers of mudanpioside (B, C, E, F, J) are found in all species, with mudanpioside E being the most abundant [36,39,41,42]. Paeonidaninol A and B have been detected exclusively in *P. peregrina* [35,43]. Paeonisuffrone and its derivatives, 1-*O*-β-D-glucosylpaeonisuffrone and 1-*O*-β-D-glucosyl-8-*O*-benzoylpaeonisuffrone, are present in both *P. peregrina* and *P. lactiflora* [36,43].

Monoterpene glycosides with a *p*-menthane skeleton, such as lactinolide, lactinolide 6-*O*-β-D-glucopyranoside, palbinone, and pyrethrin I and II, are restricted to *P. lactiflora* [29,44]. Similarly, paeonilactone A, B, and C have been identified in *P. lactiflora* and *P. officinalis* [29,45,46].

In the essential oil of *P. mascula*, terpenoids such as nopinone, phytol, geraniol, tetrahydrofarnesyl acetone, *p*-cymene, and *cis*-myrtanal have been identified [47,48]. In the seed oil of *P. lactiflora*, 24-methylenecycloartanol and squalene are prevalent [49].

#### 2.1.2. Flavonoids

All five *Paeonia* species contain flavonoids (Table 1), a group of natural compounds known for their diverse pharmacological effects, such as antioxidant, anti-inflammatory, antitumor, and cardioprotective activities [50]. These include quercetin and its derivatives, such as quercetin 3-galacto-7-rhamnoside, quercetin-dihexoside, quercetin 3-*O*-β-D-galactopyranoside, quercetin 3-*O*-β-D-glucopyranoside, rutin, and others [42,49,51,52,53,54,55]. Kaempferol and its derivatives (e.g., astragalin, kaempferol 3-*O*-(6″-galloyl)-β-D-glucopyranoside, and populnin) are found in *P. tenuifolia*, *P. mascula*, and *P. lactiflora* [39,41,55,56,57]. Isoramnetin occurs in *P. peregrina*, *P. tenuifolia*, and *P. mascula* [13,41,42], while limocitrin is present in both *P. tenuifolia* and *P. lactiflora* [39,55,57]. Apigenin, known for its anti-cancer and neuroprotective effects [58,59], is identified in *P. mascula* and *P. lactiflora* [41,49], while *P. lactiflora* also contains malvoside, naringenin, taxifolin glucosides, and liquiritin apioside [60,61,62].

An important subgroup of flavonoids is the anthocyanins, which exhibit strong antioxidant, antitumor, neuroprotective, and anti-inflammatory properties [63]. Anthocyanins are present in all *Paeonia* species except *P. mascula* (Table 1). These include petunidin, cyanidin and its glucoside, peonidin and glucosides, delphinidin derivatives, malvidin, and others [13,29,39,52,64,65].

#### 2.1.3. Phenolic Acids

Phenolic acids are present in all five *Paeonia* species (Table 1) and contribute significantly to their health-promoting properties, including potent radical-scavenging activities. The most abundant phenolic acid is gallic acid and its derivatives—methyl gallate, digallic acid, ethyl gallate, galloylglucose, and phenylethanol gallate—which are found in both the petals and roots of *Paeonia* species [7,13,29,39,41,42,51,53,66,67]. Chlorogenic and vanillic acids have been identified in the seed oil of *P. lactiflora* [49], while *p*-hydroxybenzoic acid is present in both *P. peregrina* and *P. tenuifolia* [22,37]. These phenolic acids are widely recognized for their antioxidant, anti-inflammatory, and potential chemopreventive effects, further enhancing the therapeutic value of *Paeonia* species [68].

#### 2.1.4. Tannins

Tannins are present in all *Paeonia* species except *P. tenuifolia* (a species that remains poorly studied phytochemically, Table 1). Among the five species, *P. lactiflora* exhibits the highest abundance of tannins, likely due to its status as the most extensively investigated species. Notable tannin compounds identified in *P. lactiflora* include tri-, tetra-, and penta-galloyl derivatives such as galloyl glucose, galloylquinic acid, casuariin, pedunculagin, strictinin, and tellimagrandin I [29,61,66]. Similar derivatives have also been detected in *P. mascula* [41,51] and *P. officinalis* [53,67]. In contrast, *P. peregrina* contains only tannic acid as a representative tannin [69]. These tannins are known for their diverse pharmacological activities, including antioxidant, antimicrobial, and astringent properties, highlighting their therapeutic significance [70].

#### 2.1.5. Other Phenolic Compounds

Paenol, a phenolic compound typical for genus *Paeonia*, is found in *P. peregrina*, *P. officinalis*, *P. tenuiflia*, and *P. lactiflora* (Table 1) [39,42,45,66,71]. Moreover, it has been found in both petals and roots and is connected with the antioxidant pharmacological activity of peonies [72]. Paeonoside and apiopaeonoside are present in the petals of *P. peregrina*, *P. officinalis*, and *P. tenuifolia* [39,42], while resveratrol and viniferin are identified in *P. mascula* [41]. The essential oil of *P. mascula* contains salicylic derivatives—salicylaldehyde and methyl salicylate [48,73].

#### 2.1.6. Lipids

Lipids have been identified in the seed oil of *Paeonia* species, specifically in *P. mascula* and *P. lactiflora* [49,74]. The sterols β-sitosterol, campesterol, stigmasterol, isofucosterol, citrostadienol, Δ7-avenasterol, and daucosterol are found in the seed oil of *P. lactiflora* [29,49]. Additionally, fatty acids such as oleic acid (mono-unsaturated), palmitic acid (saturated), and linoleic acid and linolenic acid (poly-unsaturated) have been identified in the seed oil of *P. mascula* [74]. These lipids contribute to the nutritional and therapeutic potential of the seed oils, with sterols often recognized for their cholesterol-lowering properties and fatty acids providing essential nutrients for health [75,76].

#### 2.1.7. Organic Acids

Organic acids have been reported in all *Paeonia* species, except *P. mascula* (Table 1). Benzoic acid has been identified in all species, though in different organs, including *P. peregrina*, *P. officinalis*, *P. tenuifolia*, and *P. lactiflora* [7,22,45,49,66,77]. Citric acid and shikimic acid are found in the petals of *P. peregrina* and *P. tenuifolia* [13,39]. *Paeonia lactiflora* is noted as a source of oleanolic acid and ursolic acid [78], while quinic acid is present in *P. peregrina*, *P. officinalis*, and *P. tenuifolia* [42]. These organic acids contribute to the antioxidant, anti-inflammatory, antimicrobial, and other therapeutic properties of *Paeonia* species, enhancing their medicinal potential [79,80,81].

#### 2.1.8. Alkaloids

Alkaloids, such as oxindole and 5-hydroxyquinolin, are found only in *P. lactiflora* (Table 1) [82]. However, they have also been reported in *P. officinalis* and *P. peregrina*, although they have not been specifically examined in these species [10,83].

**Table 1:** Bioactive compounds of peonies.

*Paeonia* Species	*P.* *peregrina*	*P. officinalis*	*P. tenuifolia*	*P.* *mascula*	*P.* *lactiflora*
Compounds					
**Terpenoids**					
(+)-Paeonilactone B	[13]				
(*Z*)-(1*S*,5*R*)-β-Pinen-10-yl β-vicianoside		[7]			
1-*O*-β-D-Glucosyl-8-*O*-benzoylpaeonisuffrone					[84]
1-*O*-β-D-Glucosylpaeonisuffrone					[84]
24-Methylenecycloartanol					[49]
4-*O*-Methyl-paeoniflorin					[84]
6′-*O*-Galloyl desbenzoyl paeoniflorin	[13]				
6′-*O*-13-D-glucopyranosylalbiflorin					[85]
6′-*O*-Benzoylalbiflorin					[85]
8-Debenzoylpaeonidanin					[40]
8-Debenzoylpaeoniflorin					[29]
Albiflorin	[13,42]	[42,86]	[42,51]	[87]	[29,36,66,85,87]
Benzoylalbiflorin		[86]			
Benzoyloxypaeoniflorin				[41]	
Benzoylpaeoniflorin	[13,35,37,43]	[86]		[41]	[29,36,84,85,87]
*cis*-Myrtanal				[48]	
Desbenzoylpaeoniflorin				[41]	
*E*-Phytol				[47]	
Galloyl paeoniflorin	[13,42]	[7,42]	[42]	[41]	[87]
Galloylalbiflorin				[41]	
Geraniol				[47]	
Hexadecanoic acid				[48]	
Isobenzoylpaeoniflorin					[84]
Isopaeoniflorin					[84]
Lactiflorin		[7]			[36]
Lactinolide					[44]
Lactinolide 6-*O*-β-D-glucopyranoside					[44]
Methyldesbenzoylpaeoniflorin				[41]	
Methylpaeoniflorin				[41]	
Mudanpioside B	[42]	[42]	[42]		
Mudanpioside C					[36]
Mudanpioside E	[42]	[42]	[42,51]	[41]	[36]
Mudanpioside F			[51]		
Mudanpioside J	[42]	[42]		[41]	
Nopinone				[48]	
Oxypaeoniflorin	[13,42]	[42,86]	[51]	[41]	[36,87]
Paeonidanin	[35,37]				
Paeonidanin A				[38]	[29,36]
Paeonidanin B					[29,36]
Paeonidanin C					[29,36]
Paeonidanin D					[29,36]
Paeonidanin E					[29,36]
Paeonidaninol A	[35,43]				
Paeonidaninol B	[35,43]				
Paeoniflorigenone	[13,37]	[71]	[52]		
Paeoniflorin	[13,35,42,43]	[7,42,86]	[39,42,51]	[41,51]	[29,36,66,84,85,87]
Paeonilactone A					[29,46]
Paeonilactone B					[29,46]
Paeonilactone C		[86]			[29,46]
Paeonin A					[40]
Paeonin B	[13]		[51]		[40]
Paeonin C					[40]
Paeonisuffrone	[43]				
Palbinone		[86]			[29]
*p*-Cymene				[47]	
Pyrethrin I					[29]
Pyrethrin II					[29]
Squalene					[49]
Texahydrofarnesyl acetone				[47]	
*Z*-Phytol				[47]	
**Flavonoids**					
(−)-Epicatechin-3-O-gallate					[60]
(+)-Catechin-3-O-β-D-glucopyranoside					[60]
(+)-Catechin-7-O-gallate					[60]
(2*R*)-(−)-Naringenin-5-O-β-D-glucopyranoside					[60]
(2*R*)-Naringenin-7-O-β-D-glucopyranoside					[60]
(2S)-(−)-Naringenin-5-O-β-D-glucopyranoside					[60]
3,5-Di-*O*-β-D-glucopyranoside			[65]		
6-Hydroxykaempferol			[51]		
Apigenin				[41]	[49]
Astragalin (Kaempferol 3-glucoside)				[41]	
Catehin					[60,66]
Cyanidin	[69]		[51]		
Cyanidin 3,5-diglucoside		[65]	[65]		[65]
Cyanidin 3-glucoside			[65]		[65]
Cyanidin 3-*O*-rhamnoside	[13]		[51]		
Delphinidin	[13]		[51]		
Delphinidin 3-glucoside	[69]				
Delphinidin 3-*O*-rhamnoside			[51]		
Foeniculin			[54]		
Hispidulin				[41]	
Isorhamnetin	[13,42]		[42,51]	[41]	
Kaempferol				[41]	[49]
Kaempferol 3,7-β-D-diglucoside					[56]
Kaempferol 3-*O*-(6″-galloyl)-β-D-glucopyranoside					[55]
Kaempherol 3-*O*-(2″-galloyl)-β-D-glucopyranoside					[55]
Lactifloraoside					[55]
Limocitrin			[51]		
Limocitrin-3-*O*-yl β–Dsophoroside			[57]		
Limocitrinyle 3-*O*-β-D-sophoroside					[55]
Liquiritin apioside					[61]
Luteolin				[41]	[49]
Malvidin	[69]				
Malvoside					[62]
Methoxy-kaempferol				[41]	
Methylarbutin					[55]
Onopordin			[51]		
Pelargonidin					
Pelargonidin 3-Glucoside		[64]	[64]		[64]
Peonidin					
Peonidin 3,5-di-*O*-β-D-glucopyranoside		[65]	[65]		[65]
Peonidin 3-glucoside		[65]	[65]		[65]
Peonin (Peonidin-3,5-diglucoside)					[29]
Petunidin	[13]				
Petunidin 3-*O*-rhamnoside			[51]		
Populnin (Kaempferol-7-*O*-glucoside)					[56]
Quercetin	[13]	[42]	[42]		[49]
Quercetin 3-galacto-7-rhamnoside	[52]				
Quercetin 3-*O*-(6″-galloyl)-β-D-glucopyranoside					[55]
Quercetin 3-*O*-β-D-Galactopyranoside			[54]		
Quercetin-3-*O*-glucoside				[51]	
Quercetin-dihexoside		[53]			
Rutin					[49]
Sexangulareinyle 3-*O*-β-D-sophoroside					[55]
Sexangularetin-3-*O*-yl β–D-sophoroside			[57]		
Taxifolin-3-*O*-β-D-glucopyranoside					[60]
**Phenolic acids**					
Chlorogenic acid					[49]
Digallic acid	[13]	[53]			
Dihydroxybenzoic acid	[13]				
Ellagic acid	[13]		[51]		
Ethyl gallate	[13]				
Ferulic acid			[51]		
Gallic acid	[13,37,42]	[42]	[24,42,51]	[41,51]	[29,66]
Galloylglucose		[29,65]			
Galloyl-norbergenin	[13]				
Methyl gallate	[13,42]	[7,42]	[42,51]	[41]	[66]
*p*-Coumaric acid	[13]		[51]		[49]
Phenylethanol gallate	[13]				
*p*-Hydroxybenzoic acid	[37]		[24]		
Vanillic acid					[49]
**Tannins**					
Tannic acid	[69]				
1,2,3,4,6-Pentagalloylglucose					[29]
1,2,3,4,6-Penta-*O*-galloyl-β-D-glucose (PGG)		[65]			[66]
1,2,3,6-Tetra-*O*-galloyl-β-D-glucose					[29]
1,2,3-Tri-*O*-galloyl-β-D-glucose					[29]
1,2,6-Tri-*O*-galloyl-β-D-glucose					[29]
1,3,6-Trigalloyl-β-D-glucose					[29]
1-*O*-Galloyl-β-D-glucose					[29]
2,3-*O*-(*S*)-HexahydroxydiphenoylD-glucopyranose					[29]
3-*O*-Digalloyl-1,2,4,6-tetra-*O*-galloyl-β-D-glucose		[65]			
3-*O*-Galloylquinic acid					[29]
4-*O*-Galloylquinic acid					[29]
5-Desgalloylstachyurin					[29]
Casuarictin					[29]
Casuariin					[29]
Hexagalloyl glucose				[51]	
Pedunculagin					[29]
Pentagalloyl glucose				[51]	
Strictinin					[29,61]
Tellimagrandin I		[86]			[29]
Tetragalloyl glucose				[41,51]	
Tetra-galloyl-hexoside		[53]			
**Other phenolic compounds**					
Apiopaeonoside	[13]				
Carashipenol A				[41]	
Methyl salicylate				[48]	
Paeonol	[13,42]	[42,71,86]	[42]		[66]
Paeonoside	[13,42]	[42]	[42]		
Resveratrol				[41]	
Salicylaldehyde				[48,73]	
Viniferin				[41]	
**Lipids**					
△7-Avenasterol					[49]
13-Methyl tetradecanoic acid					[29]
Campesterol					[49]
Citrostadienol					[49]
Daucosterol					[29]
Isofucosterol					[49]
Linoleic acid				[74]	
Linolenic acid				[74]	
Oleic acid				[74]	
Palmitic acid				[74]	
Stigmasterol					[49]
α-/δ-Тocopherol					[49]
β-Sitosterol					[29,49]
β-Sitosterol 3-O-β-D-glucopyranoside		[7]			
**Organic acids**					
Benzoic acid	[24]	[7,86]	[24]		[49,66,77]
Citric acid	[13]		[51]		
Oleanolic acid					[78]
Picrocrocinic acid	[13]				
Quinic acid	[42]	[42]	[42]		
Shikimic acid	[13]		[51]		
Ursolic acid					[78]
**Alkaloids**					
Oxindole					[82]
5-Hydroxyquinoline					[82]

### 2.2. Pharmacological Properties of Peonies

The pharmacological properties of the five peony species are studied at different extent of precision [2,7,8,13,29,39,46,47,48,49,50,51,52,53,56,66,72,74,76,83,88,89,90,91,92,93,94,95,96,97,98,99,100,101,102,103]. The main activities are summarized in Table 2. All studied species possess antioxidant and antimicrobial activity. Anti-inflammatory activity is reported for *P. peregrina* and *P. lactiflora*, while wound-healing activity is documented for *P. peregrina*, *P. officinalis*, and *P. tenuifolia.* Neuroprotective activity has been poorly studied.

#### 2.2.1. Antioxidant Activity

Extracts from various parts (leaves, petals, roots, seeds) of all five *Paeonia* species demonstrate strong antioxidant activity, primarily associated with phenolic compounds, particularly in *P. officinalis*, *P. peregrina*, and *P. mascula* [7,13,29,39,48,49,51,53,55,72,89,93,94]. A detailed summary of the methods and results of the antioxidant assays of the Paeonia species is given in Table 3. Variations in antioxidant potency are influenced by differences in methods, species, plant parts, and solvents used [72]. Additionally, synergistic effects between the diverse bioactive compounds may further enhance the overall antioxidant activity. The ability of *Paeonia* species to effectively scavenge reactive oxygen species (ROS) and combat oxidative stress highlights their potential in treating various human diseases associated with oxidative damage, including atherosclerosis, neurodegeneration, inflammation, diabetes, and aging. Consequently, there is growing interest in utilizing antioxidants as a preventive strategy for these conditions [94].

#### 2.2.2. Antimicrobial Activity

Various levels of antimicrobial activity have been demonstrated across the five *Paeonia* species [7,13,29,39,42,47,72,89,90,91]. Minimal inhibitory concentration (MIC) against various pathogens is shown in Table 4. Extracts from *P. officinalis* have been shown to exhibit greater potency compared to those from *P. peregrina* and *P. mascula* using the agar well diffusion method. The ethyl acetate extract of *P. officinalis* has been reported to effectively inhibit the Gram-positive bacteria *Listeria monocytogenes* and *Staphylococcus aureus* with 18 mm of inhibition zone; the Gram-negative bacteria *Pseudomonas aeruginosa* and *Escherichia coli* with 24 and 21 mm of inhibition zone, respectively; and lastly, the fungus *Candida albicans* with 18 mm of inhibition zone. Notably, the extract has a stronger impact on Gram-negative bacteria than on Gram-positive bacteria [72].

Additionally, hydroalcholic extract from *Paeonia officinalis* roots has demonstrated antimicrobial activity against *Bacillus cereus* (Minimal Bactericidal Concentration of 1mg/mL and a MIC of 0.5–2 mg/mL), while the roots’ ethyl acetate fractions II showed antibacterial potential against *Klebsiella pneumoniae* (MIC of 0.43 mg/mL) [7,46].

*Paeonia peregrina* petals have been tested against Gram-positive bacteria *Staphylococcus aureus* and *S. lugdunensis*, as well as Gram-negative bacteria *Proteus vulgaris*. Methanol extract obtained by maceration has been shown to be effective against *S. lugdunensis* (MIC 0.0625 mg/mL); conversely an ultrasound-assisted extract displays an MIC of 0.25 mg/mL in both *P. vulgaris* and *S.* aureus. Among the fungal species, the greatest antifungal impact was observed against *C. albicans* (MIC of 0.125 mg/mL) from the petal methanolic extract [13].

*Paeonia tenuifolia* aqueous extracts have been tested against the same pathogens, showing an MIC of 125 mg/mL in *S. lugdunensis* and *P. vulgaris*, and an MIC of 0.25 mg/mL in *S. aureus. P. tenuifolia* methanolic extract has been shown to be up to four times less effective against *C. albicans* compared to *P. peregrina* [13,39].

Two compounds from the roots of *Paeonia lactiflora* with significant concentrations, tellimagrandin I and 1,2,3,4,6-pentagalloylglucose (Table 1), exhibit antibacterial pathway activity, with 1,2,3,4,6-pentagalloylglucose being the most abundant. This compound can reduce bacterial growth by limiting fatty acid biosynthesis, while tellimagrandin I can reduce drug resistance to antibiotics and enhance the antibacterial action of the administered antibiotic [29].

The essential oil from the whole plant of *Paeonia mascula* shows antibacterial activity against *Yersinia pseudotuberculosis* and *B. Cereus* with inhibition zones of 6 and 7 mm, respectively, but no activity is observed against *E. coli*, *P. aeruginosa*, *L. monocytogenes*, *S. aureus*, and *Enterococcus faecalis*, as well as the yeast-like fungus *Candida tropicalis* [47].

Microorganisms have the ability to form polymicrobial aggregates, known as biofilms. In most biofilms, microorganisms constitute less than 10% of the dry mass, while the matrix can account for over 90%. This matrix consists of various biopolymers, referred to as extracellular polymeric substances, which are responsible for surface adhesion and cohesion within the biofilm [95]. Petal extracts of *P. peregrina* and *P. tenuifolia* have been tested for their antibiofilm activity against *Staphylococcus lugdunensis* [13,39]. Only the methanol extract of *P. peregrina* petals shows antibiofilm activity with a percentage of inhibition of 14% at 0.0156 mg/mL (1/4 of MIC), though it is much less potent than the methanolic and aqueous petal extracts of *P. tenuifolia* (79% at an MIC value of 0.5 mg/mL) [13,39].

#### 2.2.3. Neuroprotective Activity

As previously mentioned, neuroprotective activity is associated with protection from ROS, which is why it is expected to be present in all peony species. A study on the antioxidant and neuroprotective properties of several peony species revealed that seed ethanolic extracts of *P. tenuifolia* and *P. mascula* demonstrate in vitro neuroprotective activity by inhibiting acetylcholinesterase (AChE, 70.66 ± 1.26% and 65.48 ± 0.79% at 50 µg/mL, respectively), butyrylcholinesterase (BChE, 17.62 ± 2% and 38.86 ± 0.98% at 50 µg/mL, respectively), and tyrosinase (TYRO, 25.87 ± 3.76% and 27.28 ± 1.37% at 50 µg/mL, respectively) [74]. All extracts displayed a concentration-dependent inhibition against all three enzymes tested and the extracts showed a strong inhibition against AChE and BChE at similar or higher levels than that of the reference (galanthamine, 90 ± 1.70% at 50 µg/mL), while they also had marked inhibitory effects toward TYRO at a comparable ratio to that of the reference (α-kojic acid, 78.89 ± 0.09 at 100 µg/mL) used for this enzyme. They also tested the active compound paeonol for its inhibitory effect against the enzymes and the results pointed out that paeonol had remarkable BChE (60.68 ± 1.93% at 200 µg/mL) and moderate AChE (37.20 ± 2.01% at 200 µg/mL) and TYRO (34.81 ± 2.30% at 200 µg/mL) inhibition [74].

These three enzymes are linked to the pathogenesis of Alzheimer’s and Parkinson’s diseases, and their inhibition is considered a therapeutic strategy for these conditions [96,97].

#### 2.2.4. Anti-Inflammatory Activity

Extracts from *P. peregrina* petals, collected from various regions of Serbia, showed significant variation in anti-inflammatory activity, attributed to differences in phenolic content compared to ibuprofen used as a reference drug [13].

*Paeonia lactiflora* has been used in traditional Chinese medicine for over 1000 years to treat pain, inflammation, and immune disorders [85]. The total glucosides of paeony (TGP), a preparation derived from its glucosides, was approved by China’s FDA in 1998 for rheumatoid arthritis treatment [33]. Paeoniflorin, the main glycoside in TGP, has strong anti-inflammatory and immune-regulatory effects, inhibiting inflammation in autoimmune disease models [85]. Additionally, *P. lactiflora* root extract reduces pro-inflammatory mediators, particularly in lung inflammation in cystic fibrosis patients [72]. Its anti-inflammatory effects are also linked to oleanolic and ursolic acids [73].

#### 2.2.5. Wound-Healing Activity

Different aqueous and methanolic petal extracts from *P. peregrina* were tested for their wound-healing capacity—an important process for skin defense and regeneration [13]. *P. peregrina* petal extracts were not toxic to keratinocytes and enhanced their migration and proliferation as well as wound contraction and epithelialization rate. This indicates that *P. peregrina* petals may aid in healing skin wounds [13].

In addition, aqueous petal extract from *P. tenuifolia* obtained by microwave-assisted extraction exhibits a significant migration capacity of about 26.14 ± 0.04% [13,39].

It was reported that extracts from different plants of the genus *Paeonia* possess enzyme-inhibitory potential with respect to both AChE/BuChE and tyrosinase, finding that the extract of *P. officinalis* is more active than *P. peregrina* [42]. The anti-tyrosinase activity exerted by *P. officinalis* extracts suggests a possible use as a skin-protective agent. In addition, it was reported that *P. officinalis* extract increases the viability of human epidermal keratinocytes while enhancing mitochondrial activity under oxidative stress [92,93].

#### 2.2.6. Anti-Cancer Activity

Chloroform extracts from the roots of two cultivars of *P. officinalis* were tested on several normal and cancer cell lines (HeLa, MCF7, and I407). These extracts did not exert any appreciable cytotoxic activity after 24h of treatment when exposed to different concentrations ranging from 1.25 μg/mL to 250 μg/mL. In contrast, the extracts induced a proliferation decrease of about 20% in ovarian carcinoma (IGROV1) and osteosarcoma cells (U2OS) already at 2.5 μg/mL by inducing a hyperpolarization of mitochondria and an increase in ROS levels, without inducing cell death [45].

The capacity of dietary phytochemicals to raise ROS generation in tumor cells and inhibit tumor cell growth has been well-documented. Increased ROS has also been shown to trigger a signaling cascade that ultimately results in cellular stress in tumor cells [101,102]. Aqueous and methanolic extracts of *P. mascula* (from 5 μg/mL to 75 μg/mL) were tested for anticancer activity by using HELA cells. The levels of several ROS inducers such as phospho-NF-kβ p65, advanced glycation end product (AGE), and BCL-2 were evaluated. Results show that *P. mascula* extracts decrease significantly the levels of ROS inducers, providing scientific basis for the pharmacological activities of *P. mascula* extracts [51].

Studies of the antitumor activity of *P. lactiflora* have shown that some compounds (peoniflorin, peanol, and 1,2,3,4,6-pentagalloylglucose) can arrest cell growth and sensitize target cells to chemotherapy drugs [29].

#### 2.2.7. Other Activities

In rats with tetrachloromethane (CCl_4_)-induced hepatopathy, Ahmad et al. found that the aqueous extract of *P. officinalis* roots, administered at 100 and 200 mg/kg/die for 14 days, had a hepatoprotective action [83]. The authors observed a general amelioration of biochemical parameters related to liver health and an improved regenerative activity, relating these effects to alkaloids and terpenes, in particular paeoniflorin and its isomer albiflorin (Table 1) [2,83]. The hepatoprotective effect of *P. lactiflora* was attributed to the content of oleanolic and ursolic acids, which are also considered the reason for its antibacterial activity [78]. Aqueous and methanolic leaf and root extracts of *P. officinalis* were reported to possess an inhibitory activity on the enzyme α-amylase—responsible for the breakdown of complex polysaccharides into disaccharides—in a range of concentration of 1.25–5 mg/mL, an effect that could prolong overall carbohydrate digestion time, therefore causing a reduction in postprandial hematic glucose levels [53]. Furthermore, methyl gallate and galloyl paeoniflorin show an important antiplasmodial activity against *P. falciparum* D6 with IC_50_ 1.57 μg/mL and IC_50_ 4.72 ng/mL, respectively, representing promising antimalarial agents [7].

Several components (TGP, paeoniflorin and 8-debenzoylpaeoniflorin, gallic acid, and paeonol) of *P. lactiflora* have been shown to modulate blood glucose via effects on glucose uptake and insulin release, or indirectly by regulating carbohydrate absorption and metabolism with respect to in vitro and in vivo systems [29]. Additionally, oleanolic and ursolic acids are related to the antidiabetic effect of this plant [78].

The anti-ulcer effect of *P. lactiflora* was tested on a HCl/ethanol-inducedgastric ulcer mouse model [66]. The protection percentage was calculated after comparison with the ulcer control group, which was considered to have 100% damage. The administration of HCl/ethanol produces lesions on the gastric mucosa, which are significantly reduced by 88.8% in animals pretreated with a 100 mg/kg extract [66].

Interestingly, the hydroalcholic root extract of *P. officinalis* administered twice daily at a dose of 20 mg/kg was found to efficiently control seizures in children with medically intractable epilepsy [8].

#### 2.2.8. Toxicity

*P. lactiflora* has no obvious toxicity [2]. Paeoniflorin, obtained from the root of this species, has low acute toxicity, but demonstrates a sedative effect in mice [3]. Additionally, the threshold of toxicological concern with respect to *P. lactiflora* root extract was found safe for use in cosmetics at a 1% concentration [103]. Although *P. peregrina* is not tested much for toxicity, it has been experimentally demonstrated that its petal extracts are not toxic to skin cells [13]. *P. officinalis* extracts are considered safe because they do not cause mortality in rats at doses of 175 mg/kg, 550 mg/kg, and 2000 mg/kg [2]. Paeonol is considered nontoxic at doses of 300 mg/kg, 2000 mg/kg, and 5000 mg/kg [2].

## 3. Materials and Methods

### 3.1. Plant Objects

*Paeonia officinalis* L.

A herbaceous perennial plant with solitary terminal flowers, *Paeonia officinalis*, known as the common peony, is widespread in areas of Southern Europe, including regions like Spain, France, Italy, and the Northwestern Balkans, excluding Bulgaria and Greece [19,20]. The *P. officinalis* group includes 6 accepted subspecies that usually reach a height of 60 to 80 cm [20,98,99]. Common features shared by the subspecies include glossy, dark green, deeply lobed leaves that are divided into segments, as well as pink/red, showy flowers that are made up of soft, delicate petals that encircle a cluster of bright yellow stamens that attract butterflies and bees [100]. Renowned for its stunning blossoms and medicinal properties, *P. officinalis* has been cultivated and cherished for centuries.

*Paeonia peregrina* Mill.

*Paeonia peregrina* is a herbaceous perennial plant with atypical rhizome. Some of the roots are spindle-shaped and thickened in a tuberous manner. The stems are unbranched, usually bearing a single flower at the top. The leaf blade is divided into two or more leaflets, which often have bristles along veins above. The petals are obovate, with some toothed at the top, and are dark or light red to pink or orange (Figure 1) [23]. This species is found at altitudes ranging from 0 to 1500 m, usually in limestone habitats in Albania, Bulgaria, Greece, Italy, Macedonia, Moldova, Romania, Serbia, and Turkey [23,101].

*Paeonia mascula* (L.) Mill.

*Paeonia mascula* is a perrenial herbaceous plant with a relatively wide distribution, ranging from Northern Spain (Cantabria and Soria) to Iraq via France, Italy, the Balkans, Cyprus, and Turkey [101]. In Bulgaria, it is a rare plant, found in a few limestone grassland habitats in several low mountains [23]. Its roots are carrot-shaped and its leaves are ovate or oblong, with petals that are pink, red, white or white with pink shade.

*Paeonia tenuifolia* L.

*Paeonia tenuifolia* is a perennial herbaceous plant with solitary terminal flowers distributed in Armenia, Azerbaijan, Bulgaria, Georgia, Romania, Russia (in the Caucasus), Serbia, Turkey (in the European part), and Ukraine. It grows in the lowlands below an altitude of 0–900 m [101]. In Bulgaria, it is a rare plant, appearing in limestone grassland habitats in several lowlands [23]. It is a very distinct peony species because of its great number of fine filamentous leaflets/leaf segments.

*Paeonia lactiflora* Pallas

*Paeonia lactiflora* is a perennial herbaceous plant. It has distinctive features such as its flowers, which are are usually several per shoot, both terminal and axillary. The leaflets and segments adaxially have bristles along the veins. Also, the roots are thick and attenuate toward tip. This species is native to China, Manchuria, Mongolia, Japan, Korea, and Russia (Far East, Siberia, and Primorye) and occurs in woods and grasslands at altitudes of between 400 and 2300 m [102]. This plant is widely cultivated in regions with temperate or cool climate, including Europe, Asia, and North America [25,26].

### 3.2. Data Search Strings

We accessed Scopus, Web of Science, PubMed, and Google Scholar to identify publications related to the phytochemistry and pharmacology of five *Paeonia* species. The search strings were “*P. peregrina*”, “*P. officinalis*”, “*P. tenuifolia*”, “*P. mascula*”, and “*P. lactiflora*”, combined with terms such as “composition”, “bioactive compounds”, “phytochemistry”, “pharmacology”, and “pharmacological effects”. The records were assessed for eligibility, following the PRISMA 2000 guidelines, and 35 publications with inappropriate entries were excluded.

We selected 44 publications that provided a comprehensive list of the chemical components of the five *Paeonia* species and 33 publications detailing their pharmacological properties. Using the published results, a dataset was created in Microsoft Excel, encompassing the phytochemistry and pharmacology of *P. peregrina*, *P. officinalis*, *P. tenuifolia*, *P. mascula*, and *P. lactiflora.* These tables were used to compare the chemical composition of the species and evaluate the relationship between their phytochemistry and pharmacological effects.

## 4. Conclusions

Our review highlights the phytochemical diversity of *P. peregrina*, *P. officinalis*, *P. tenuifolia*, *P. mascula*, and *P. lactiflora*. The bioactive compounds identified across these species include monoterpene glycosides, flavonoids, and phenolic acids. Due to limited research, other phytochemicals such as tannins, lipids, and organic acids have been sparsely reported or remain unidentified, particularly in *P. mascula* and *P. tenuifolia*.

All five species exhibit strong antioxidant and antimicrobial properties, which vary depending on habitat, plant parts, extraction, and methods of analysis. Their antioxidant activity likely contributes to other pharmacological effects, including neuroprotective, anti-inflammatory, anti-cancer, wound-healing, and hepatoprotective properties.

The review further reveals that *P. officinalis* and *P. lactiflora* have been more extensively studied than *P. peregrina*, *P. tenuifolia*, and *P. mascula*, both in terms of chemical composition and pharmacological properties. This research gap presents an opportunity for further studies and points toward the necessary tests.

In general, this review underscores *P. lactiflora* as the species with the highest number of identified bioactive compounds and pharmacological activities. Given the vulnerable and protected status of *P. peregrina*, *P. tenuifolia*, and *P. mascula* in Bulgaria and the limited research on these native and rare species, *P. lactiflora* could be well recommended as a substitute for medicinal use. This popular Chinese peony is not only well known for its medicinal properties, it is also vastly cultivated as an ornamental plant. Therefore, the replacement of *P. peregrina* roots and petals gathered from wild populations with plant substances derived from cultivated *P. lactiflora* is not only feasible but also highly recommended as a sustainable alternative.

## Figures and Tables

**Table 2:** Pharmacological activities of peonies.

*Paeonia* Species	*P.* *peregrina*	*P.* *officinalis*	*P.* *tenuifolia*	*P.* *mascula*	*P.* *lactiflora*
Activity					
Antioxidant activity	[13,72]	[7,53,89]	[39,88]	[48,51]	[29,49,56]
Antimicrobial activity	[13,72]	[7,46]	[39]	[47,90]	[29,91]
Neuroprotective activity			[74]	[74]	
Anti-inflammatory activity	[13]				[29,77]
Wound-healing activity	[13]	[46,100]	[39]		
Anti-cancer activity		[49,101,102]		[51]	[29]
Others		Hepatoprotective [2,83] Anti-diabetic activity [53] Antimalaria effect [7] Seizure control in children with medically intractable epilepsy [8]	50		Glycemic activity [29] Anti-ulcer effect [66]

**Table 3:** Antioxidant activity of peonies.

*Paeonia* Species	*P. peregrina*	*P. officinalis*	*P. tenuifolia*	*P. mascula*	*P. lactiflora*
Reference	[13,72]	[53]	[39,88]	[48,51]	[53]
Antioxidant activity	ABTS^•+^—26–36 mmol TE/g DPPH^•^ IC_50_—0.110–0.125 mg/mL CUPRAC—0.29–0.38 mol TE/g FRAP—80–230 μmol Fe^2+^/g	ABTS^•+^—886.0–4610 μM TE/g DWE DPPH^•^—343.4–2553 μM TE/g DWE ORAC—1232–1433 μmol TE/g DWE HOSC—1957–2012 μmol TE/g DWE HORAC—1566–1891 μmol CAE/g DWE	ABTS IC_50_—0.070–0.099 mg/mL DPPH IC_50_—0.051–0.126 mg/mL CUPRAC—0.319–0.391 mol TE/g FRAP—559.5–849.4 μmol Fe^2+^/g ABTS—66.99–69.15% DPPH IC_50_—85.12–90.11%	DPPH—46.11–58.21% NO—43.4–56.9%	ORAC—2635.71 μmol TE/100 g ABTS—1582.82 μmol TE/100 g FRAP—447.92 μmol TE/100 g DPPH 359.55 μmol TE/100 g

ABTS—2,2′-Azinobis-3-Ethylbenzthiazolin-6-Sulfonic Acid; DPPH—2,2-Diphenyl-1-picrylhydrazyl; CUPRAC—Cupric Reducing Antioxidant Capacity; FRAP—ferric reducing antioxidant power; ORAC—oxygen radical absorbance capacity; HORAC—Hydroxyl Radical Antioxidant Capacity.

**Table 4:** Antimicrobial activity—minimal inhibitory concentration (MIC mg/mL) of peony extracts.

*Paeonia* Species	*P.* *peregrina*	*P.* *officinalis*	*P.* *tenuifolia*	*P.* *mascula*	*P. lactiflora*
Reference pathogenic bacteria	[13,72]	[7,46]	[39]	[47,90]	[91]
*Bacillus cereus*	0.125–4	0.5–2	0.5–4	7	-
*Escherichia coli*	0.5–4	0.5–2	0.5–2	-	1.25
*Klebsiella pneumoniae*	-	0.43	-	5.65	1.25
*Listeria monocytogenes*	0.5–4	0.25–2	1–4	-	-
*Proteus vulgaris*	0.25–2	-	-	-	-
*Pseudomonas aeruginosa*	0.5–4	0.25–2	0.5–4	-	-
*Salmonella typhimurium*	0.5–4	1–2	0.5–4	-	0.62
*Staphylococcus* *lugdunensis*	0.0625–1	-	-	-	-
*Staphylococcus aureus*	0.25–4	1–2	1–4	9.30	1.25

## Data Availability

Not applicable.
